# Postnatal adaptations of phosphatidylcholine metabolism in extremely preterm infants: implications for choline and PUFA metabolism

**DOI:** 10.1093/ajcn/nqaa207

**Published:** 2020-08-10

**Authors:** Kevin C W Goss, Victoria M Goss, J Paul Townsend, Grielof Koster, Howard W Clark, Anthony D Postle

**Affiliations:** Child Health, Academic Unit of Clinical and Experimental Sciences, Faculty of Medicine, University of Southampton, Southampton, United Kingdom; NIHR Southampton Respiratory Biomedical Research Unit, University Hospitals Southampton, Southampton, United Kingdom; NIHR Southampton Respiratory Biomedical Research Unit, University Hospitals Southampton, Southampton, United Kingdom; NIHR Southampton Respiratory Biomedical Research Unit, University Hospitals Southampton, Southampton, United Kingdom; NIHR Southampton Respiratory Biomedical Research Unit, University Hospitals Southampton, Southampton, United Kingdom; Child Health, Academic Unit of Clinical and Experimental Sciences, Faculty of Medicine, University of Southampton, Southampton, United Kingdom; NIHR Southampton Respiratory Biomedical Research Unit, University Hospitals Southampton, Southampton, United Kingdom; Child Health, Academic Unit of Clinical and Experimental Sciences, Faculty of Medicine, University of Southampton, Southampton, United Kingdom; NIHR Southampton Respiratory Biomedical Research Unit, University Hospitals Southampton, Southampton, United Kingdom

**Keywords:** preterm infants, plasma phosphatidylcholine, stable isotopes, CDP-choline pathway: PEMT pathway

## Abstract

**Background:**

Lipid metabolism in pregnancy delivers PUFAs from maternal liver to the developing fetus. The transition at birth to diets less enriched in PUFA is especially challenging for immature, extremely preterm infants who are typically supported by total parenteral nutrition.

**Objective:**

The aim was to characterize phosphatidylcholine (PC) and choline metabolism in preterm infants and demonstrate the molecular specificity of PC synthesis by the immature preterm liver in vivo.

**Methods:**

This MS-based lipidomic study quantified the postnatal adaptations to plasma PC molecular composition in 31 preterm infants <28 weeks’ gestational age. Activities of the cytidine diphosphocholine (CDP-choline) and phosphatidylethanolamine-*N*-methyltransferase (PEMT) pathways for PC synthesis were assessed from incorporations of deuterated *methyl*-D_9_-choline chloride.

**Results:**

The concentration of plasma PC in these infants increased postnatally from median values of 481 (IQR: 387–798) µM at enrollment to 1046 (IQR: 616–1220) µM 5 d later (*P* < 0.001). Direct incorporation of *methyl*-D_9_-choline demonstrated that this transition was driven by an active CDP-choline pathway that synthesized PC enriched in species containing oleic and linoleic acids. A second infusion of *methyl*-D_9_-choline chloride at day 5 clearly indicated continued activity of this pathway. Oxidation of D_9_-choline through D_9_-betaine resulted in the transfer of 1 deuterated methyl group to *S*-adenosylmethionine. A very low subsequent transfer of this labeled methyl group to D_3_-PC indicated that liver PEMT activity was essentially inactive in these infants.

**Conclusions:**

This study demonstrated that the preterm infant liver soon after birth, and by extension the fetal liver, was metabolically active in lipoprotein metabolism. The low PEMT activity, which is the only pathway for endogenous choline synthesis and is responsible for hormonally regulated export of PUFAs from adult liver, strongly supports increased supplementation of preterm parenteral nutrition with both choline and PUFAs.

See corresponding editorial on page 1417.

## Introduction

Extremely preterm infants, with immature respiratory, digestive, and immunological systems, have a substantially different overall metabolism and physiology compared with older children and adults. While significant advances in neonatal care have improved their survival (1, 2), detailed information is lacking about many of these metabolic differences. Preterm delivery interrupts the normal placental transfer and fetal accretion of choline (3), complex fatty acids (4, 5), and phospholipids and fats (6) that occur in the third trimester. Extremely preterm infants cannot tolerate hyperosmolar or high-volume enteral feeds and must be supported parenterally for several days or weeks (7). An improved understanding of how preterm infants metabolize their parenteral nutrition is required, and this study focuses on metabolic adaptations of phosphatidylcholine (PC) metabolism in the immediate postnatal period in a cohort of extremely preterm infants, using stable isotope labeling.

Choline is an essential nutrient (8) required for synthesis of PC, sphingomyelin, and acetylcholine. It is an important source of methyl groups and impacts on DNA/RNA methylation and on the folate, homocysteine, methionine, and vitamin B-12 metabolic pathways. Pregnancy is a time of high choline demand (9, 10) with active transport across the placenta depleting maternal supplies, leading to a recent recommendation that pregnant women should increase dietary choline intakes above currently advised amounts (6). PC derived from the hepatic maternal phosphatidylethanolamine-*N*-methyltransferase (PEMT) pathway, enriched in PUFA-containing species containing arachidonic acid(20:4n–6) (AA) and DHA, is selectively transported to the fetus in the third trimester (6). Plasma choline concentration is relatively high in the developing fetus and in term infants, 6–7 times greater than adult values (11). High concentrations of choline in human breast milk (12) ensure an adequate supply of choline in the breastfed term infant and choline supplementation of preterm infants is required to preserve fetal concentrations of plasma choline in the perinatal period (13).

**FIGURE 1:**
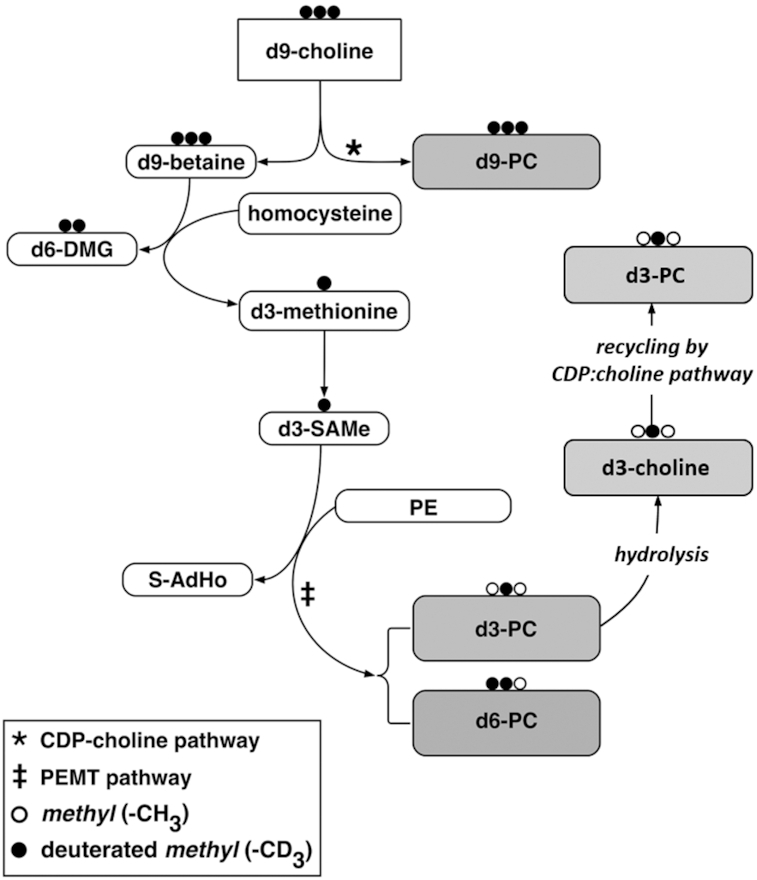
Metabolic pathway of intravenously administered choline and its methyl groups using deuterium-labeled *methyl*-D_9_-choline. Methyl groups labeled with deuterium are represented by the black dots; the unlabeled methyl groups are represented by the white dots. The *methyl*-D_9_-choline can enter the CDP-choline pathway (*) to produce D_9_-PC. Alternatively, it can be oxidized to D_9_-betaine, which then donates a single methyl group to homocysteine, producing D_6_-DMG and D_3_-methionine. D_3_-methionine serves as a precursor for D_3_-SAMe, which can be used by PEMT (‡) to sequentially methylate PE, forming D_3_-PC as well as smaller amounts of D_6_-PC. A fraction of D_3_-choline or D_6_-choline is released by hydrolysis of PEMT-derived D_3_-PC or D_6_-PC in the liver and recycled back to D_3_-PC or D_6_-PC by the CDP-choline pathway. CDP-choline, cytidine diphosphocholine; DMG, dimethylglycine; PC, phosphatidylcholine; PE, phosphatidylethanolamine; PEMT, phosphatidylethanolamine *N*-methyltransferase; S-AdHo, *S*-adenosylhomocysteine; SAMe, *S*-adenosylmethionine.

Direct incorporation of choline into PC by the cytidine diphosphocholine (CDP-choline) pathway generates mainly monounsaturated and di-unsaturated molecular species (14). The alternative PEMT pathway, which synthesizes PUFA-enriched PC by 3 sequential methylations of phosphatidylethanolamine (PE) (**Figure 1**) (15), is the sole means of endogenous choline production and accounts for ∼30% of total liver PC synthesis (16) in adults. Stable isotope labeling with *methyl*-D_9_-choline chloride, combined with electrospray ionization tandem MS (ESI-MS/MS), has been used to quantify the synthesis and turnover of individual molecular species of PC in lung surfactant in human volunteers (17), of hepatic PC in vivo (16), and in adult patients ventilated for acute respiratory distress syndrome (18). Here, we have applied this technology to characterize PC metabolism and turnover in preterm infants and have demonstrated the molecular specificity of PC synthesis by the immature preterm liver in vivo, monitored by the appearance of label in plasma PC.

## Methods

### Study participants

A total of 31 infants were recruited between February 2010 and December 2011 from the Neonatal Unit at Princess Anne Hospital, Southampton, United Kingdom (**Table 1**). Intubated and ventilated infants (≤48 h old, ≤1250 g weight, and ≤28 wk and 6 d gestation at birth) were eligible for recruitment. Exclusion criteria included extreme cardiovascular instability requiring multiple inotropes, significant pulmonary hemorrhage, persistent air leak, an identified severe congenital abnormality, or if the infant was unlikely to survive between *methyl*-D_9_-choline administration and initial sample collection. Infants (*n* = 31) were recruited over a 2-y period and comprised 45% (14/31) males with a median birth weight of 720 g (IQR: 670–945 g) and a median gestation of 25 wk and 5 d (IQR: 24+2–26+4 wk). A total of 62% (19/31) were born by vaginal delivery and 29% (9/31) were breech presentation. Overall mortality was 19.3% (6/31), with a median birth weight of 620 g (IQR: 599–686 g), median gestation of 24 wk 0 d (IQR: 23+5–24+2 wk), and time of death at a median postnatal age of 10 d (IQR: 4.75–20.5 d). The main cause of death was severe lung disease that was unresponsive to treatment (*n* = 5) and intraventricular hemorrhage (*n* = 1). All infants born after 25 weeks of gestation survived. The majority (90.3%, 28/31) of infants received intravenous total parenteral nutrition (TPN) before baseline samples were collected. Only 9.6% (3/31) of the recruited infants received >24 mL · kg^−1^ · d^−1^(nontrophic/nutritive volumes) of enteral feeds by 120 h. The majority of infants were therefore receiving TPN for all of their energy and nutritional needs during the initial phase of the study. The lipid source of the TPN was ClinOleic (Baxter Healthcare), which is a mixture of 80% olive oil and 20% soybean oil, comprising largely MUFAs. The composition of ClinOleic and the TPN is shown in **Supplemental Table 1**. This table also summarizes the choline and methionine concentration in the TPN. The mean intake of energy, protein, carbohydrate, and choline over the first week of postnatal life is summarized in in **Supplemental Figure 1**.

**TABLE 1:** Characteristics of infants in the study^1^

Gender	Male (*n* = 14)	Female (*n* = 17)
Gestational age, d	179 ± 10	
Range	164–202	
Birth weight, g	781 ± 159	
Range	545–1095	
Survival, no. of infants who died	6	

^1^Values are means ± SDs unless otherwise indicated.

The Southampton and South West Hampshire Research Ethics Committee (A) approved the study (reference 09/H0502/95), and written informed consent was obtained from parents or guardians.

### Study design

This was an observational cohort study of extremely premature infants, requiring neonatal intensive care, who were administered 3.6 mg/kg of *methyl-*D_9_-choline chloride intravenously over 3 h within 48 h of preterm birth. Management decisions relating to all aspects of clinical care, including the timing and administration of TPN, were decided by attending neonatologists and were recorded by the research team. Those infants who remained intubated and ventilated at 120 h after recruitment (10/31; 32%) received a second dose of *methyl-*D_9_-choline chloride to monitor metabolism of the repeated bolus. In order to reduce any analytical bias, all of the MS and data analyses were blinded to the identities of all the infants in the study. The number of study participants was determined empirically, as there was no previous comparable data available.

### Sample collection and processing

Baseline EDTA venous blood samples of 250 µL were collected before the *methyl-*D_9_-choline chloride infusion, and then at 6, 12, and 24 h, and subsequently every 24 h, up tod after infusion. The antioxidant butylhydroxytoluene (200 µL of 1 mg/mL in 0.9% NaCl) was added to whole-blood samples, which were then centrifuged at 3000 × *g* at 4°C for 15 min with supernatants frozen at −80°C for analysis.

### Measurement of plasma phospholipids and isotopic enrichments

Aliquots (100 µL) of thawed plasma were lipid extracted using methanol and chloroform (19) after adding 10 nmol of the internal standard dimyristoylPC. The lower organic phase was dried under nitrogen gas at 37°C and stored at −80°C for analysis by ESI-MS/MS. PC species were analyzed by direct infusion (shotgun lipidomics) using a Waters Xevo TQ mass spectrometer (Waters) equipped with an ESI interface. Lipid extract samples were dissolved in 200 µL solvent (methanol/dichloromethane/water/concentrated ammonia; 7:2:0.8:0.2 vol:vol) and introduced to the mass spectrometer by syringe driver at a flow rate of 10 µL/min. Unlabeled PC (D_0_-PC) species were quantified from precursor ion scans of the protonated phosphocholine fragment (*m/z* + 184) and D_9_-PC synthesized via the CDP-choline pathway from corresponding scans of *m/z* + 193 (20). Precursor scans of *m/z* + 187 (D_3_-PC) or +190 (D_6_-PC), containing 1 or 2 recycled deuterium-labeled methyl groups identified products of the PEMT pathway (21). Isotope enrichments in PC were calculated from the following formula:

(1) $$\begin{eqnarray*}
&& {\rm{\% \ enrichment\ }}\nonumber\\
&&\quad = {\rm{ \Sigma D}}9{\rm{\ or\ \Sigma D}}3{\rm{*}}100\,/\left( {{\rm{\Sigma D}}0 + {\rm{ \Sigma D}}3 + {\rm{\Sigma D}}6 + {\rm{ \Sigma D}}9} \right)
\end{eqnarray*}$$

Spectra were processed by MassLynx 4.1 (Waters), exported to Microsoft Excel, and analyzed by in-house Visual Basic macro algorithms.

### Mass isotopomer distribution analysis of flux via PEMT

The flux through the PEMT pathway was calculated using mass isotopomer distribution analysis (MIDA) (22, 23) to determine the enrichment of the D_3_–*S*-adenosylmethionine (-SAMe) pool in the liver at each time point (16). The formula for D_3_-SAMe enrichment (%) was as follows:

(2) $$\begin{eqnarray*}
&& SAMe{\rm{\ }}\left( {\rm{\% }} \right)\nonumber\\
&& = \left( {\frac{{\Sigma \left( {abundance{\rm{\ }}of{\rm{\ }}{D_6}PC} \right)}}{{\Sigma \left( {abundance{\rm{\ }}of{\rm{\ }}{D_3}PC + abundance{\rm{\ }}of{\rm{\ }}{D_6}PC} \right)}}} \right){\rm{\ *}}100\nonumber\\
\end{eqnarray*}$$

D_3_-PC enrichments were corrected by hepatic D_3_-SAMe enrichments to provide estimates of PC synthesis by PE-*N* methylation, expressed as the percentage of total PC or individual PC species synthesized by that pathway.

### Statistical analysis

Statistical analysis was conducted using IBM SPSS Statistics version 21 (IBM Corporation), and figures were generated by GraphPad Prism 8.2.1. Multiple comparisons were performed using ANOVA and the Kruskal-Wallis test. Paired concentration comparisons were analyzed using the Mann-Whitney test and composition comparisons by 2-tailed *t* test. Numbers of infants decreased steadily with time both due to mortality and to extubation and subsequent withdrawal from the study.

## Results

### Phospholipid profile at birth and through the first 5 d

**FIGURE 2:**
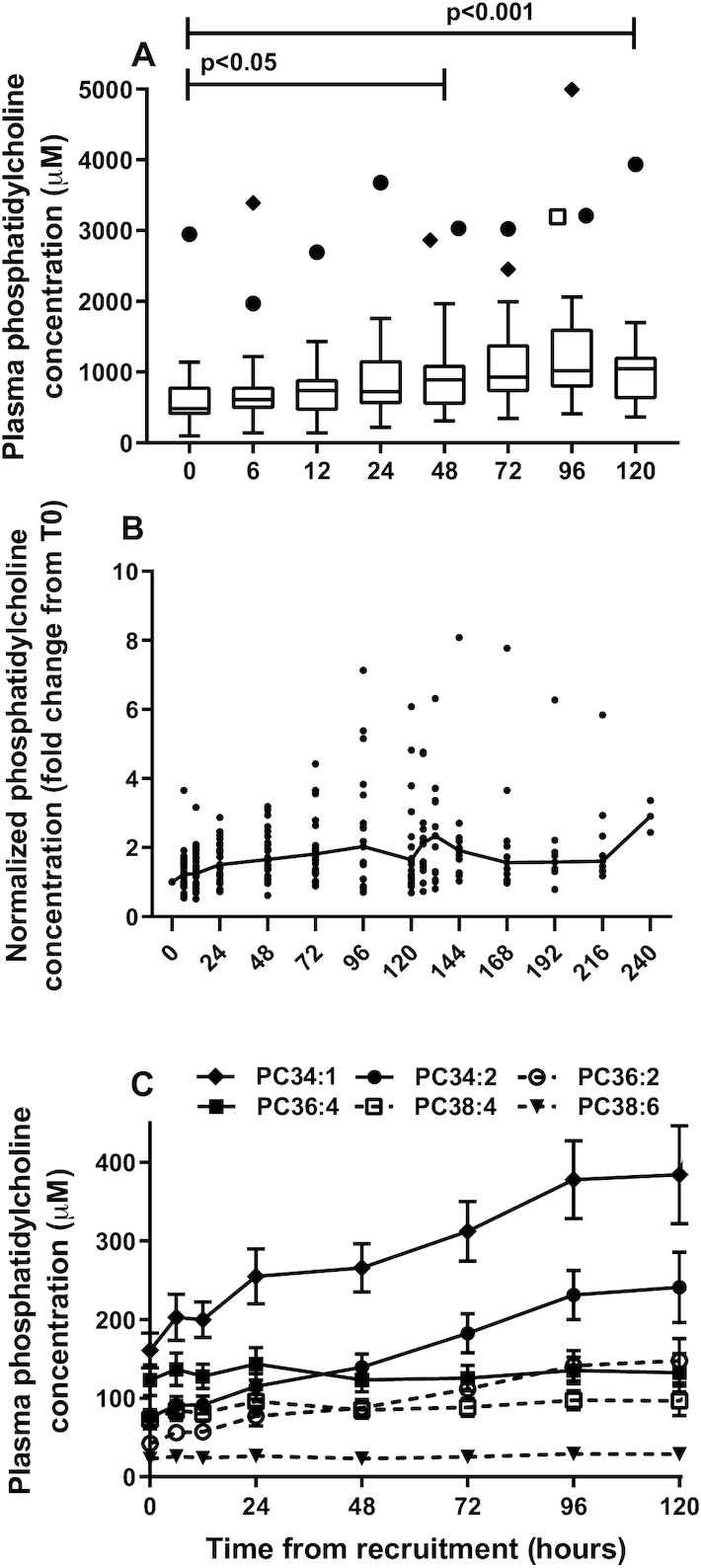
Concentration of plasma PC in preterm infants. (A) PC concentration increased with time from recruitment and exhibited considerable variation. Results are shown as a box-and-whisker plot with medians, IQRs. The symbols above the box-and-whisker plots represent outlier values. Each symbol refers to 1 of 3 of the same infants who had high concentrations of plasma PC. Significance was assessed using the Mann-Whitney test. (B) PC concentration from time of recruitment for individual infants, expressed relative to the initial value at *t* = 0 h. (C) Concentrations of selective PC molecular species over the first 5 d of the study. Results are expressed as means ± SEMs. Numbers of infants decreased at each time point from 31 at *t* = 0 to 23 at *t* = 120 h. PC, phosphatidylcholine.

Plasma PC concentration was calculated from the sum of all molecular species >1% of the total ion count of the diagnostic scan for unlabeled PC in >50 of samples analyzed (*n* = 284). Concentrations of the total PC species selected (*n* = 15) over the first 5 d of the study are detailed in **Supplemental Table 2**. Corresponding ions were selected for comparison in the various scans for stable isotope–labeled PC species. Total plasma PC concentration at recruitment in this cohort of preterm infants was lower (median: 481.7 µM; IQR: 387–798 µM) (**Figure 2**A) than reported values for adult plasma (1.94 ± 0.19 mM) (18). Plasma PC concentration increased significantly over the first 5 d of the study to 1046 µM (IQR: 616–1220 µM) (*P* < 0.001), but this masked a considerable variation between individual infants (Figure 2B). All values in this figure have been normalized to the plasma PC concentration of the initial sample (*t* = 0 h). While plasma PC concentration increased by an order of magnitude for some infants, it remained virtually constant for others. However, this fractional increase did not correlate either with gestational age or with severity of neonatal chronic lung disease (nCLD).

**FIGURE 3:**
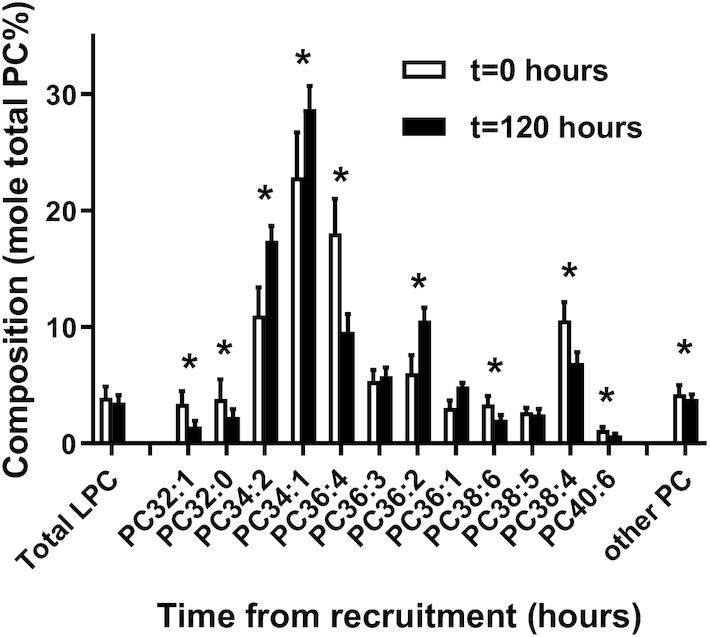
PC molecular species composition of preterm infant plasma. Results are presented at recruitment and expressed as a percentage of total PC (*t* = 0 h, *n* = 31, open bars) and at 5 d (*t* = 120 h, *n* = 23, closed bars). Values are means ± SDs. *P < 0.001 (*t* test). LPC, lysophosphatidylcholine; PC, phosphatidylcholine.

The contribution of individual PC molecular species to this measured increase in total PC was calculated for an illustrative subset of 6 species. The most abundant PC species was PC 34:1 (predominantly oleoyl-containing PC 16:0/18:1), which increased from 160.5 ± 123.9 µM at *t* = 0 to 384.3 ± 293.1 µM at *t* = 120 h (Figure 2C). Concentrations of PC 34:2 and 36:2 (predominantly linoleoyl species PC 16:0/18:2 and PC 18:0/18:2) also increased steadily over the first 5 d of the study. By contrast, plasma concentrations of PUFA-containing species PC 36:4, 36:6, 38:4, and 38:6 (predominantly the AA species PC 16:0/20:4 and PC 18:0/20:4 and the DHA species PC 16:0/22:6 and PC 18:0/22:6, respectively) remained essentially constant over these 5 d. While the overall change to the molecular specificity of plasma PC reflects the transition from placental to postnatal nutrition, the precise details of the changes observed may also have been related to administration of TPN. This is suggested from analysis of plasma PC in the 3 infants from whom baseline samples were collected before initiation of TPN (**Supplemental Figure 2**). All linoleoyl-containing PC species increased substantially within 24 h of administration of TPN. Analysis in concentration terms can mask changes in composition, illustrated by the compositions of plasma PC at recruitment and at *t* = 120 h (**Figure 3**). This figure clearly shows the significant (*P* < 0.001) proportional increased contribution of mono- and di-unsaturated PC species (PC 34:1, PC 34:2, PC 36:2) and decreased fractional amounts of PUFA-containing species (PC 36:4, PC 38:4, PC 38:6, PC 40:6).

### Incorporation of stable isotope label into total plasma PC

**FIGURE 4:**
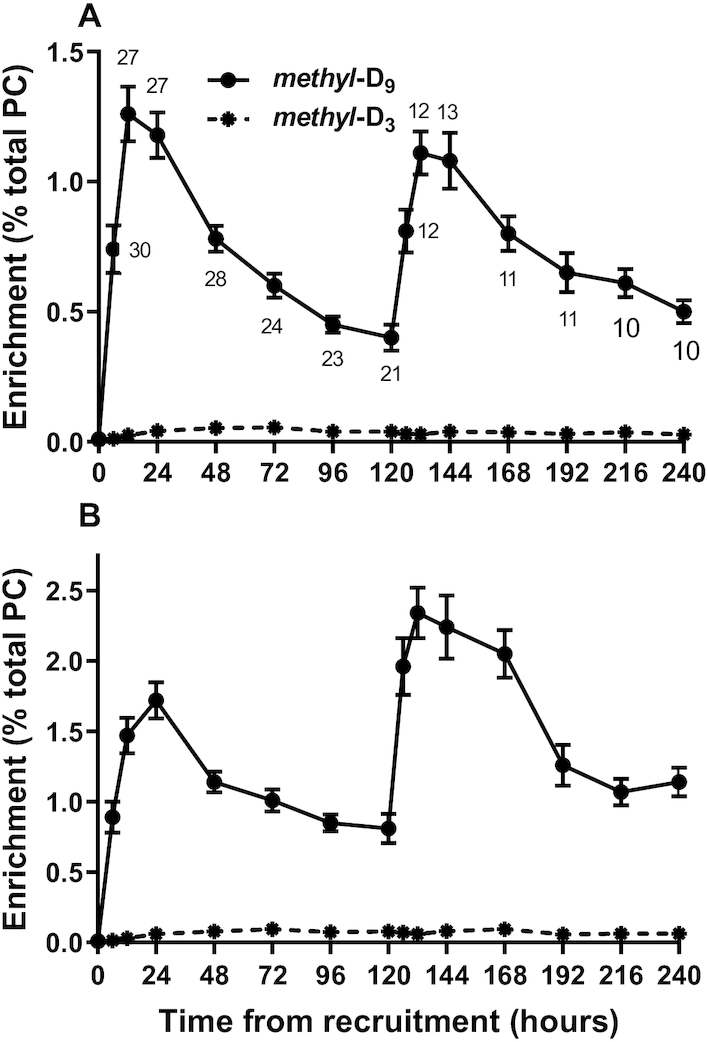
Incorporation of stable isotope–labeled choline into plasma PC in preterm infants. (A) The fractional enrichment of *methyl*-D_9_-PC (solid line, mean ± SEM) synthesized by the CDP-choline pathway and of *methyl*-D_3_-PC synthesized by the PEMT pathway (dashed line, mean with SEM smaller than symbol). There were 31 infants at *t* = 0, and numbers of infants at each time point are indicated on the figure. (B) The same results as in panel A after normalization to the initial concentration value using the variation in PC concentration with time detailed in Figure 2B. CDP-choline, cytidine diphosphocholine; PC, phosphatidylcholine; PEMT, phosphatidylethanolamine *N*-methyltransferase.

The postnatal concentration increase in selected plasma PC species significantly complicated analysis of the incorporation of *methyl-*D_9_-choline into D_9_-PC, as decreased enrichment would, in part, be due to increased concentration. By contrast, an equilibrium condition is a prerequisite for calculation of product turnover from precursor incorporation in typical stable isotope–labeling studies. Consequently, incorporation results into total plasma PC are presented both as straightforward enrichment values (**Figure 4**A) and after normalizing such enrichments for the increased plasma PC concentration (Figure 4B).

#### CDP-choline pathway

Fractional incorporation of *methyl-*D_9_-choline into D_9_-PC was rapid, reaching a maximum enrichment of 1.26% ± 0.55% at 12 h after the start of the infusion, which was equivalent to a mean enrichment rate of 0.11%/h (Figure 4A). The rate of *methyl*-D_9_-choline incorporation into PC for individual patients did not correlate with either gestational age or birth weight. As we have previously demonstrated in mice (15) that the appearance of labeled PC in plasma is a direct reflection of liver PC synthesis, these results represent a high activity of the CDP-choline pathway in the livers of preterm infants. Enrichment normalization, however, in effect provides pseudo-equilibrium conditions (Figure 4B) and demonstrates a maximum effective enrichment of 1.72% ± 0.67% at 24 h, which is comparable to the time scale of maximal enrichment in adult volunteers between 24 and 36 h (18). The incorporation rate of label into PC by the CDP-choline pathway for infants who received a second dose of *methyl*-D_9_-choline at 120 h was essentially identical to that of the complete cohort after the first dose. The normalization correction indicated that the magnitude of the additional incorporation above the value at 120 h (2.34–1.53 = 1.53%) was identical to that after the first dose, demonstrating maintained hepatic synthesis of plasma PC.

#### PEMT pathway

PC species with a single deuterated methyl group (D_3_-PC), derived from D_3_-SAMe, were used to quantify synthesis through the PEMT pathway. In contrast to the rapid incorporation through the CDP-choline pathway, the fractional incorporation of D_3_-methyl groups into PC species was lower and gradual over the first 72 h (Figure 4A). Maximum enrichment by the PEMT pathway, at 72 h, was 1/23rd (0.056% ± 0.044% compared with 1.26% ± 0.55%) of the maximal incorporation from the CDP-choline pathway at 12 h. This difference was similarly apparent after normalization for initial plasma PC concentration (Figure 4B), and after administration of the second dose of *methyl-*D_9_-choline, when maximal enrichment by the PEMT pathway of 0.094% ± 0.04% at 168 h was 1/25th of that by the CDP-choline pathway at 132 h (2.34% ± 0.62%). This low incorporation through the PEMT in contrast to the CDP-choline pathway in preterm infants was also substantially lower than previously reported for adult volunteers, where maximum enrichment of D_3_-PC was 0.32% compared with 0.51% for enrichment of D_9_-PC (18).

### Enrichment of individual PC molecular species

**FIGURE 5:**
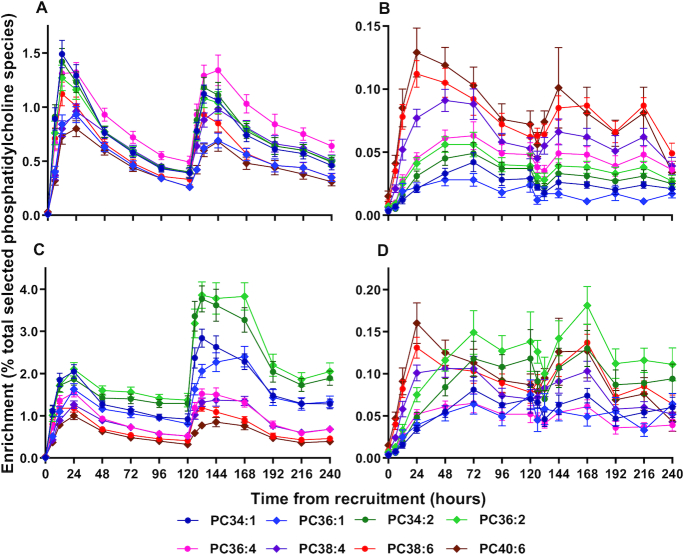
Enrichment of *methyl*-D_9_-choline into individual molecular species of plasma PC. Results are presented for incorporations into *methyl*-D_9_-PC (A, C) and *methyl*-D_3_-PC (B, D). The results in panels A and B were expressed as percentage labeled:labeled + unlabeled PC. Panels C and D present the same results after normalization to initial concentrations of individual PC molecular species at *t* = 0 h. Results are presented as means ± SEMs; numbers at each time point were the same as in Figure 4A. PC, phosphatidylcholine.

Enrichment patterns are illustrated in **Figure 5** for 4 pairs of PC molecular species containing 1, 2, 4, or 6 unsaturated double bonds, with either palmitic acid (16:0) or stearic acid (18:0) at the *sn*-1 position. Results are presented for the CDP-choline pathway (D_9_-PC) without (Figure 5A) and with (Figure 5C) correction for the relative concentration change for the respective individual molecular species compared with *t* = 0. Comparable results for the PEMT pathway with and without normalization for concentration are shown respectively in Figure 5B and D.

Normalization revealed aspects of the molecular specificity of PC synthesis by the CDP-choline pathway that were not readily apparent in the initial analysis. Without normalization, label was incorporated rapidly into all PC species, with a 2-fold greater enrichment in PC 34:1 compared with PC 40:6 (Figure 5A). While this analysis might suggest preferential enrichment of PC 36:4, taking concentration changes into account tells a very different story (Figure 5C). Normalization revealed a consistent hierarchy of synthesis over the full 240 h, with greatest enrichment of di-unsaturated species, followed by monounsaturated and then polyunsaturated species. This analysis also shows a more gradual incorporation of label into PC species containing *sn*-1 stearate (diamond symbols) than into the corresponding *sn*-1 palmitoyl-containing species (circle symbols).

Despite the low flux through the PEMT pathway (Figure 5B), the initial incorporation pattern of label into D_3_-PC species in the preterm infants was very similar to that in adult volunteers (18). Infants exhibited preferential fractional incorporation of the *methyl*-D_3_ label into polyunsaturated PC species over the first 12 to 24 h compared with either total PC composition or to the pattern of D_9_-choline incorporation. Subsequently, the fractional incorporation into D_3_-PC was characterized by a progressive increase in mono- and di-unsaturated species. This preferential incorporation of label into polyunsaturated species was still apparent after normalization (Figure 5D). As reported previously for adult volunteers, these results demonstrated very different incorporation profiles for the 2 pathways of PC synthesis, with preferential initial synthesis of di-unsaturated species by the CDP-choline pathway and of polyunsaturated species by the PEMT pathway. The D_3_-incorporation results in Figure 5 strongly suggest recycling of choline by the liver, with initial incorporation into polyunsaturated species by the PEMT pathway, followed by their subsequent metabolism and incorporation of D_3_-choline into di-unsaturated PC species by the CDP-choline pathway. Consequently, we suggest that incorporation of the D_3_-label into a combination of PC 38:4, PC 38:6, and PC 40:6 provides a more reliable index of PEMT pathway flux than that of the total number of PC species analyzed.

### Effect of disease severity on plasma PC synthesis

**FIGURE 6:**
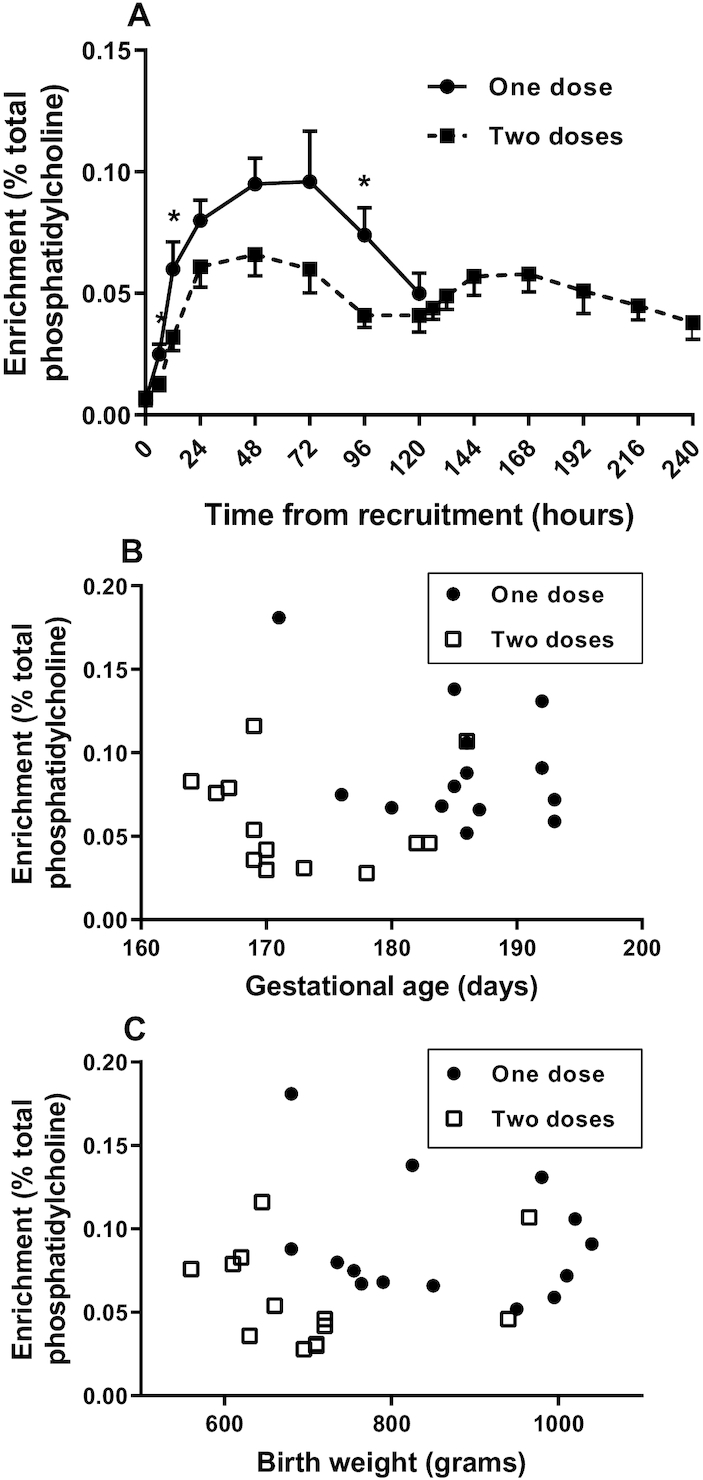
The effect of disease severity and immaturity on label incorporation into PC molecular species by the PEMT pathway. Combined enrichments of the polyunsaturated species PC 38:4, PC 38:5, PC 38:6, and PC 40:6 were calculated in panel A for infants with more severe disease who received a second dose of *methyl*-D_9_-choline at 120 h compared with infants who received a single dose of label (mean ± SEM; *P < 0.05, *t* test). Initially, 14 infants received the single dose and 13 infants received 2 doses. Corresponding numbers at 120 h were 6 and 11 infants. Infants with too few samples to calculate label incorporation (*n* = 4) were excluded. The association of PEMT, calculated from *methyl-*D_3_ label enrichment at 24 and 48 h, is shown for gestational age (B) and birth weight (C). PC, phosphatidylcholine; PEMT, phosphatidylethanolamine *N*-methyltransferase.

Disease severity, estimated by requiring ventilator support at 5 d and receiving a second dose of *methyl-*D_9_-choline chloride, had no significant effect on PC synthesis by the CDP-choline pathway. Maximal enrichments of the *methyl*-D_9_ label in PC were similar in infants who received 1 or 2 doses of the label and there was no correlation of these values with either gestational age or birth weight (results not shown). In contrast, PC synthesis by the PEMT pathway over the first 96 h was significantly lower in infants who subsequently received the second dose of label (**Figure 6**A), a result highly dependent on gestational age (Figure 6B) and birth weight (Figure 6C). Maximum enrichment of the *methyl*-D_3_ label in infants receiving the single label dose was 0.096% ± 0.069% (gestational age: 185.4 ± 6.4 d) compared with 0.066% ± 0.031% (gestational age: 172.8 ± 7.1 d) in infants receiving 2 doses (*P* < 0.001).

### Rate of synthesis by the PEMT pathway

**FIGURE 7:**
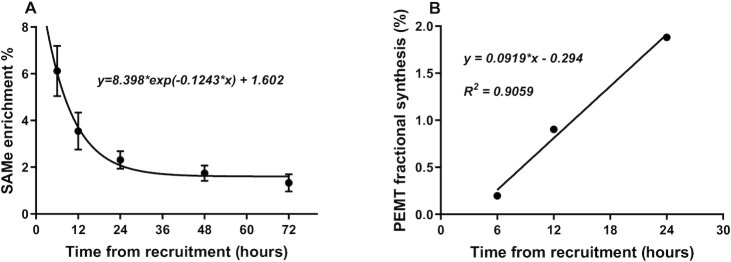
Fractional rate of plasma PC synthesis by the PEMT pathway. (A) The enrichment of hepatic SAMe was determined by MIDA calculation based on the relative incorporations of the *methyl*-D_3_ and *methyl*-D_6_ labels into PC (mean ± SEM, *n* = 27). (B) Rate of fractional synthesis by the PEMT pathway after correcting enrichment of *methyl*-D_3_-PC for enrichment of SAMe at each time point (% total PC). MIDA, mass isotopomer distribution analysis; PC, phosphatidylcholine; PEMT, phosphatidylethanolamine *N*-methyltransferase; SAMe, *S*-adenosylmethionine.

Using MIDA, flux through the PEMT pathway was calculated to determine the enrichment of the D_3_-SAMe pool in the liver (**Figure 7**A) at each time point up until a plateau was reached at 72 h. Then, these estimates of enrichment of D_3_-SAMe substrate enrichment were used to correct the corresponding D_3_-PC enrichment up to 24 h, before substantive label recycling into the CDP-choline pathway and hence representative of PEMT flux (Figure 7B). This near-linear incorporation provided an estimate of the fractional synthetic rate of PC by PE-*N* methylation that, at 0.091%/h, was considerably lower than comparable calculations for adult volunteers (0.53%/h) and mice (4.71%/h) (16). Further analysis of the synthesis of individual D_3_-PC species showed rates that varied from a maximum of 0.289%/h for D_3_-PC 40:6 to a minimum of 0.0489%/h for D_3_-PC 34:1 or a 6-fold difference in magnitude. Identical rank orders of synthesis of plasma PC species by this pathway were observed in adults, but the range of flux seen was from 2.76% to 0.25%, or 10 times higher than those seen in the preterm infant.

### Synthesis of plasma lysophosphatidylcholine

**FIGURE 8:**
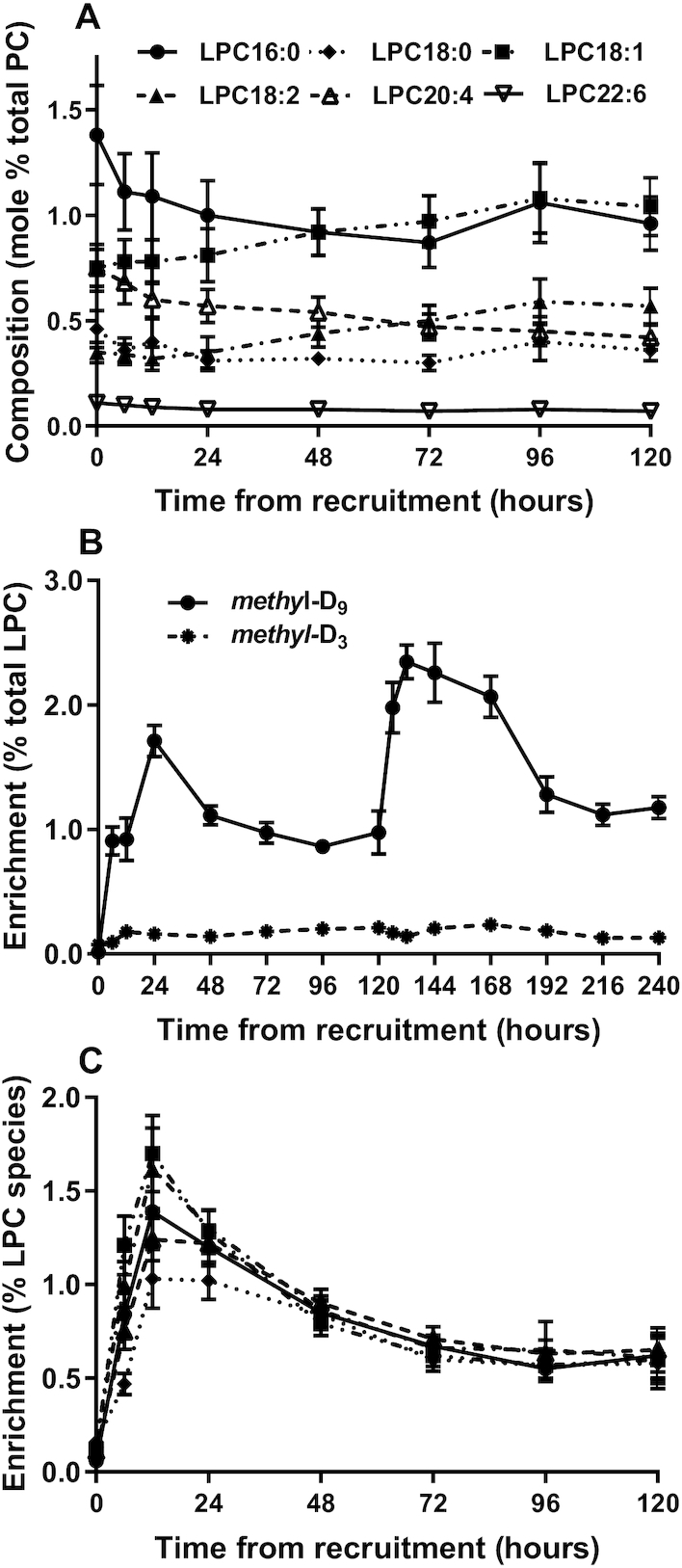
Synthesis of plasma LPC. (A) Composition of individual molecular species of plasma LPC (% total PC + LPC; mean ± SEM). (B) Enrichments of *methyl*-D_9_ (solid line) and *methyl*-D_3_ (dashed line) labels in plasma LPC species normalized to their initial concentrations at *t* = 0 h (mean ± SEM). (C) Enrichment of *methyl*-D_9_-choline in LPC species over the first 5 d after recruitment. Numbers at each time point were the same as in Figure 4A. LPC, lysophosphatidylcholine; PC, phosphatidylcholine.

Label incorporations into plasma lysophosphatidylcholine (LPC) species were evaluated to assess their suggested role as carriers of PUFAs, especially DHA, to the developing perinatal brain (24, 25). Total LPC was a consistent fraction of total PC at 3.67% ± 0.14%. In agreement with the temporal increased proportion of PC 34:1 and PC 34:2 (Figure 2C), the fractional plasma content of LPC 18:1 and LPC 18:2 increased during the course of the study, while those of LPC 16:0 and LPC 20:4 decreased (**Figure 8**A). Significantly, the content of LPC 22:6 remained very low at all time points. After normalization for the progressive increase in concentration, the kinetics of *methyl*-D_9_ and *methyl*-D_3_ label incorporations into total LPC species (Figure 8B) reflected the label incorporations into PC in terms both of time scale and magnitude (Figure 4B). Incorporation of the *methyl*-D_9_ label displayed comparable kinetics for all individual LPC species (Figure 8C). Importantly, incorporation of *methyl*-D_9_-choline into LPC 22:6 was so low that it could not be readily quantified. Similarly, *methyl*-D_3_ incorporation into individual LPC species was too low for quantification.

## Discussion

Adaptations to maternal lipid metabolism are core metabolic activities for optimal fetal development, characterized by the hyperlipidemia of pregnancy (26), increased placental lipoprotein lipase activity, and increased placental transport of fatty acids into the fetal circulation with progression of gestation. The relative roles of the fetal liver and placenta in the regulation of fetal lipoprotein metabolism have not been clear. This study presents strong evidence that the liver of preterm infants soon after birth, and by extension that of the fetal liver, is metabolically highly active in lipoprotein metabolism, demonstrated by the high activity of the hepatic CDP-choline pathway (Figure 4). The absence of a control group of infants was a consequence of extreme prematurity of these infants and is, of course, a limitation to this study. A recent study by Bernhard et al. (13) used similar methodology to show that PC synthesis by the CDP-choline pathway remained active in a more mature group of preterm infants at 3–4 weeks’ postnatal age, with continued low activity of the PEMT pathway.

Analysis of *methyl*-D_9_-choline incorporation data presented significant challenges, as the study did not conform to the equilibrium conditions typical of stable isotope incorporation studies. Consequently, we propose that normalization of our stable isotope incorporation results to the magnitude of the change in plasma PC concentration provides a more meaningful calculation of the enrichment data (Figure 4). The time scale of these normalization data (Figure 4B) was very comparable to that of the previous adult data, with maximal enrichment at 24 h and a slower decay. Importantly, the increment of the normalized incorporation for the 13 infants who received a second dose of *methyl*-D_9_-choline at 120 h was virtually identical to the initial incorporation after the first dose of labeled substrate. This observation strongly suggests that the rate of liver PC synthesis and secretion into plasma was maintained over the first week of postnatal life in extremely preterm infants.

In contrast to the PC synthesis by the CDP-choline pathway, measured activity of the PEMT pathway was very low after both *methyl*-D_9_-choline doses (Figure 4). The maximal enrichment of *methyl-*D_3_-PC to that of *methyl-*D_9_-PC was <5% in these infants compared with >60% in healthy adult volunteers (18). Possible reasons for this difference include the livers of preterm infants undergoing relatively rapid growth, as PEMT activity is low in recovery growth after partial hepatectomy (27) and in proliferating hepatoma cells (28, 29), and low estrogen activity, as PEMT induction is estrogen sensitive (30). As PEMT-dependent conversion of PE to PC is the sole mechanism for synthesis of choline, low PEMT activity indicates that extremely preterm infants are fundamentally dependent on dietary sources of choline. The concentration of circulating choline is ∼4-fold greater in the fetal compared with maternal plasma, decreases rapidly postnatally in preterm infants (31), but was maintained at near-fetal concentration by choline supplementation (13). Choline is an important source of methyl group donation and inadequate choline supply increases the risk of methylation problems in the developing preterm infant.

The low PEMT activity in these preterm infants strongly suggests that this pathway makes a minor contribution to hepatic DHA and AA supply to the developing brain in preterm infants. This conclusion implies that PUFA delivery in these preterm infants was dependent on the CDP-choline pathway, although *methyl*-D_9_ labeling of PUFA-containing PC species (especially of DHA species) was relatively low. Recent evidence suggests that combined dietary supplementation with both choline and DHA is required for hepatic export of newly synthesized DHA-PC species in TPN-fed preterm infants (13). The combination of low concentration and low *methyl*-D_9_ labeling of LPC 22:6 (Figure 8), together with undetectable *methyl*-D_3_ labeling, indicates that LPC is not a major carrier of DHA supply to the brain in preterm infants.

Molecular species analysis of PC synthesis highlights fundamental processes of adaptation of lipid metabolism to postnatal life. The greater fractional abundance of long-chain (LC)-PUFA–containing PC in the fetal compared with the maternal circulation [biomagnification (32)] is due both to adaptations to maternal hepatic PC synthesis and to selectivity of LC-PUFA delivery to the fetus (33). Three infants who received delayed TPN nutrition demonstrated the importance of postnatal nutrition to the steady increment with time of 18:2-containing PC species (Figure 2).

The greater incorporation into PC 34:2 and PC 36:2 compared with LC-PUFA–containing species (Figure 5C), given their relatively constant catabolic rates, was a direct cause of their increased concentration. Moreover, the virtually identical enrichment increments of PC 34:2 and PC 36:2 after the second *methyl*-D_9_-choline dose indicated preservation of these synthetic rates over the first week of postnatal life. Figure 5 indicates a prolonged duration of label enrichment over the time scale of this project, due to extensive recycling of the choline head group demonstrated by the *methyl*-D_3_-PC enrichment data (Figure 5D). While maximal enrichment of *methyl*-D_3_-PC was maximal for PUFA-PC species at 24 h, those of mono- and di-unsaturated species being maximal some 2 d later (Figure 5D) indicated significant recycling of the choline moiety of PC. As we reported previously for healthy adult volunteers (18), we interpret this discrepancy as the limited synthesis of *methyl*-D_3_-choline by PEMT activity entering the substrate pool for the CDP-choline pathway after hydrolysis of newly synthesized *methyl*-D_3_-PC.

While further interpretation of these results would have been helped considerably by analysis of PC distributions in plasma lipoprotein subclasses, this was precluded by the small volumes of the serial blood samples taken in this study. Increased cord blood concentrations of VLDL and LDL and HDL cholesterol have been reported in preterm compared with term infants (34), but there are no reports of changes in lipoprotein subclass concentrations over the first week of postnatal life in extremely preterm infants.

The implications for clinical care are 3-fold. First, as synthesis of DHA-PC species is not prioritized by the CDP-choline pathway, low PEMT activity has potential consequences for reduced supply of DHA to the developing postnatal brain. Failure to provide enough PUFA could therefore have a significant impact on their neurodevelopment during a period of rapid growth. Second, reduced flux through the PEMT pathway in these preterm infants places them at particular risk of choline deficiency, as they are primarily dependent on dietary sources of choline. Third, SAMe is an important methyl donor, not just for the PEMT pathway but also for other methyl transfer reactions involving proteins and nucleic acids. Methylation patterns in promoter regions of DNA play a vital role in influencing gene expression and therefore alter, by epigenetics, future disease development and health (35). This study provides additional support for supplementation of DHA and choline in TPN diets of preterm infants (13).

## Supplementary Material

nqaa207_Supplemental_File

## Notes

The National Institute for Health Research Respiratory Biomedical Research Unit provided salary support to KCWG, VMG, JPT, and GK and purchased the mass spectrometer. The Wellcome Trust provided an equipment grant (reference 057405).

Supplemental Tables 1 and 2 and Supplemental Figures 1 and 2 are available from the “Supplementary data” link in the online posting of the article and from the same link in the online table of contents at https://academic.oup.com/ajcn/.

ADP and HWC are joint senior authors on this paper.

Data described in the manuscript, code book, and analytic code will be made available upon request pending application.

Abbreviations used: AA, arachidonic acid (20:4n–6); CDP-choline, cytidine diphosphocholine; ESI-MS/MS, electrospray ionization tandem MS; LC, long-chain; LPC, lysophosphatidylcholine; MIDA, mass isotopomer distribution analysis; PC, phosphatidylcholine; PE, phosphatidylethanolamine; PEMT, phosphatidylethanolamine *N*-methyltransferase; SAMe, *S*-adenosylmethionine; TPN, total parenteral nutrition.
