# Supplementary material for: Postnatal adaptations of phosphatidylcholine metabolism in extremely preterm infants: implications for choline and PUFA metabolism
Source: Am J Clin Nutr. 2020 Aug 10;112(6):1438–47. doi: 10.1093/ajcn/nqaa207 (PMC7727469; doi:10.1093/ajcn/nqaa207)
Supplement: nqaa207_Supplemental_File [file nqaa207_supplemental_file.pdf]

Postnatal adaptations of phosphatidylcholine metabolism in extremely preterm infants: implications for choline and polyunsaturated fatty acid metabolism. Goss K.C.W. “Online Supplementary Material”

Online Supporting Material

Postnatal adaptations of phosphatidylcholine metabolism in extremely preterm infants: implications for choline and polyunsaturated fatty acid metabolism

Kevin C.W. Goss[1,2], Victoria M. Goss[2], J. Paul Townsend[2], Grielof Koster[2], Howard W. Clark[1,2] & Anthony D. Postle[1,2]

[1]Child Health, Academic Unit of Clinical & Experimental Sciences, Faculty of Medicine, University of Southampton and [2]NIHR Southampton Respiratory Biomedical Research Unit, University Hospitals Southampton, Southampton, UK

Postnatal adaptations of phosphatidylcholine metabolism in extremely preterm infants: implications for choline and polyunsaturated fatty acid metabolism. Goss K.C.W. "Online Supplementary Material"

Online Supporting Material

Supplementary Table 1 Composition of total parenteral nutrition

Fatty acid composition of ClinOleic 20%

Saturated (% total)	15
Monounsaturated (% total)	65
Polyunsaturated (% total)	20
Unsaturated fatty acid composition	
Oleate (% total)	58.3
Linoleate (% total)	17.7
Arachidonate (% total)	0.3
Linoleneate (% total)	2.0
Docosahexaenoate (% total)	0.23

Parenteral nutrition composition

	Preterm TPN	ClinOleic 20%
Energy (kcal/ml)	0.63	1.67
Protein (g/ml)	0.02	0.00
Carbohydrate (g/ml)	0.13	0.00
Fat (g/ml)	0.00	1.67
Choline (mg/ml)	0.00	1.04
Methionine (mg/ml)	0.60	0.00

Postnatal adaptations of phosphatidylcholine metabolism in extremely preterm infants: implications for choline and polyunsaturated fatty acid metabolism. Goss K.C.W. “Online Supplementary Material”

Online Supporting Material

Supplementary Table 2. Concentrations of individual molecular species of plasma phosphatidylcholine from preterm infants over the first 5 day of the study.
Results are presented as median vales with inter-quartile ranges indicated (µmoles/litre).
Formulae are described by total number of fatty acyl carbon atoms and unsaturated double bonds, with the major molecular species indicated in column 2.

PC	Major acyl	t=0h			t=6h			t=12h			t=24h			t=48h			t=72h			t=96h			t=120h		
species	composition	Median	25%	75%	Median	25%	75%	Median	25%	75%	Median	25%	75%	Median	25%	75%	Median	25%	75%	Median	25%	75%	Median	25%	75%
total		482	388	798	610	470	803	725	504	896	770	539	1171	882	539	1106	830	655	1395	1019	824	824	1044	750	1323
PC30:0	PC16:1_16:1	2.97	2.32	3.85	2.79	2.37	4.12	3.26	2.29	4.25	3.79	2.63	5.02	2.6	2.2	3.74	2.44	1.7	3.25	2.4	1.79	1.79	1.87	1.67	2.8
PC32:1	PC16:0_16:1	17.1	13.3	29.4	23.2	15.8	29.1	24.9	13.6	30.1	26.8	15.9	36.5	21.2	13.2	26.1	15	11.4	26.4	15.8	10	10	13.7	9.07	21.4
PC32:0	PC16:0_16:0	19.8	15.7	25.2	20.8	15.7	29.4	21.8	15.7	28.5	22.6	18.4	37.5	23.4	17.9	35.1	25.7	13.9	33	23.3	15.3	15.3	21.7	16.8	30.8
PC34:3	PC16:0-18:3	2.93	2.16	3.76	3.59	2.27	5.01	4.1	2.41	4.81	4.56	3.16	7.36	5.4	4.17	7.86	7.65	4.37	10.5	7.83	5.62	5.62	8.13	4.84	12
PC34:2	PC16:0_18:2	60.1	42.2	86.6	72.4	48.5	117	85.8	54.7	111	92.8	66.8	144	128	83.6	168	130	98	250	179	138	138	188	133	231
PC34:1	PC16:0_18:1	136	92.1	204	158	106	236	180	114	251	204	150	329	243	178	300	253	174	396	301	223	223	324	208	397
PC36:4	PC16:0_20:4	97.3	69.7	157	111	82.8	143	114	81	157	127	82	171	117	72.4	134	97	76.7	141	121	75.6	75.6	108	73.6	143
PC36:3	PC18:1_18:2	27.6	20.5	46.4	34.1	28.1	48.9	42.1	28.3	53.8	44.8	31.1	72.8	53.1	36.2	70.1	56.5	38.8	91.6	66.6	48.6	48.6	63	44	90
PC36:2	PC18:0_18:2	32.1	20.5	50.1	40.4	27.9	73.2	50.5	32.8	71.3	53.2	38.8	94	75.6	49.4	113	81.9	59.9	143	107	79.7	79.7	120	73.2	151
PC36:1	PC18:0_18:1	16.7	12.5	28.2	22.4	14.9	33.3	25.5	16.3	34.8	30.7	21.7	46.5	38	25.1	46.9	40.1	25.4	64.9	49.4	39.3	39.3	55.8	35.8	64.6
PC38:6	PC16:0_22:6	18.3	11.9	30.8	20.4	17.6	25.9	21.5	13.7	31.5	23.6	15.4	33.5	21.4	14.8	27.1	21.5	15	30.4	22.9	18.1	18.1	21.5	17	30.5
PC38:5	PC18:1_20:4	13.9	10.4	25.1	15.9	12.6	24.9	21.6	12.4	29.3	24.8	16.4	35.3	27.1	16.5	35.7	27.6	17.9	37.2	26.9	22.8	22.8	24.4	20.6	38
PC38:4	PC18:0_20:4	54.6	39.9	94.8	66	49.3	88.2	73.8	53.7	101	75.5	53.4	111	78	47.4	99	67	51.4	102	76.6	57.2	57.2	77.9	53.2	97.1
PC38:3	PC18:0_20:3	13.4	7.5	18	12.4	10.5	17.8	14.6	10.9	20.5	18.2	11.7	23.5	16.3	11.5	20.9	15.7	10.9	21.1	17.7	12.9	12.9	17.6	11	23.2
PC40:6	PC18:0_22:6	6.25	4.29	10.4	6.97	5.84	9.63	7.98	5.45	10.9	8.41	5.53	12.4	7.48	5.46	10.1	7.19	5.07	10.3	8.05	6.29	6.29	7.09	6.11	10.4

Postnatal adaptations of phosphatidylcholine metabolism in extremely preterm infants: implications for choline and polyunsaturated fatty acid metabolism. Goss K.C.W. “Online Supplementary Material”

Online Supporting Material

Supplementary Figure 1. Daily nutrient intakes (mean +S.D.) were calculated over the first week of postnatal life using the Southampton Electronic Neonatal Nutrition Assessment Tool (SENNAT) (1).

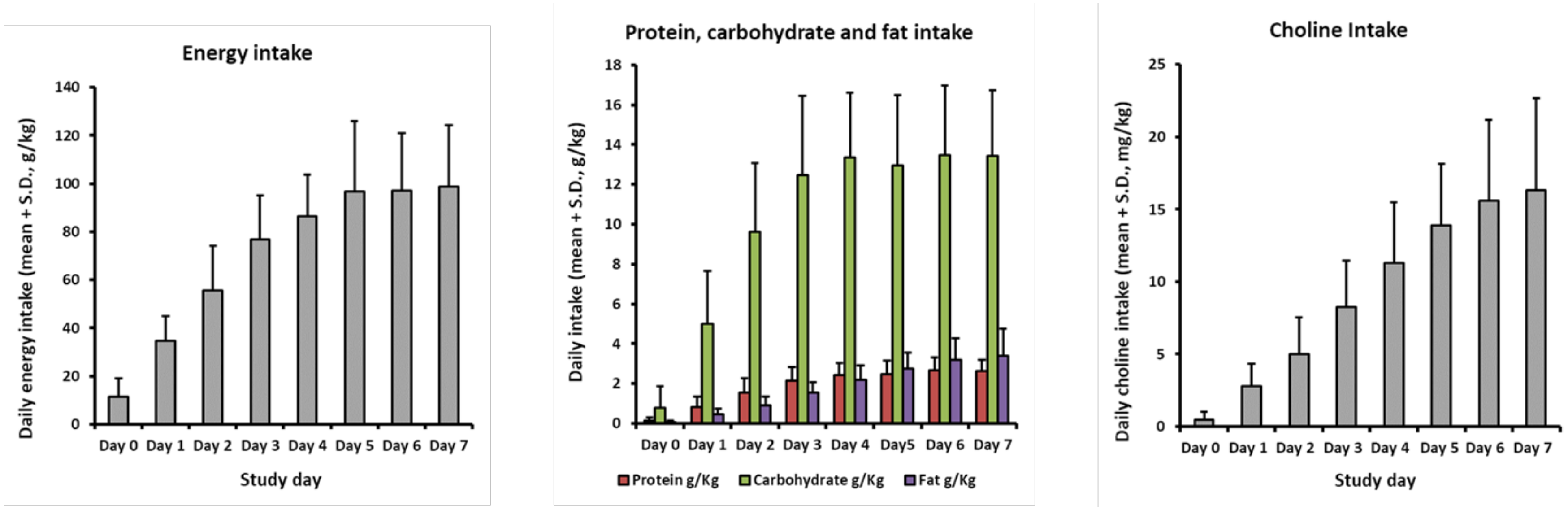

Postnatal adaptations of phosphatidylcholine metabolism in extremely preterm infants: implications for choline and polyunsaturated fatty acid metabolism. Goss K.C.W. "Online Supplementary Material"

Online Supporting Material

Supplementary Figure 2. Relative proportions of selected molecular species of plasma phosphatidylcholine for the three infants who started administration of TPN within the time frame of the study. The time of TPN administration for each infants is indicated by the black arrow. The fractional content of PC species containing 18:2 increased after initiation of TPN for all three infants.

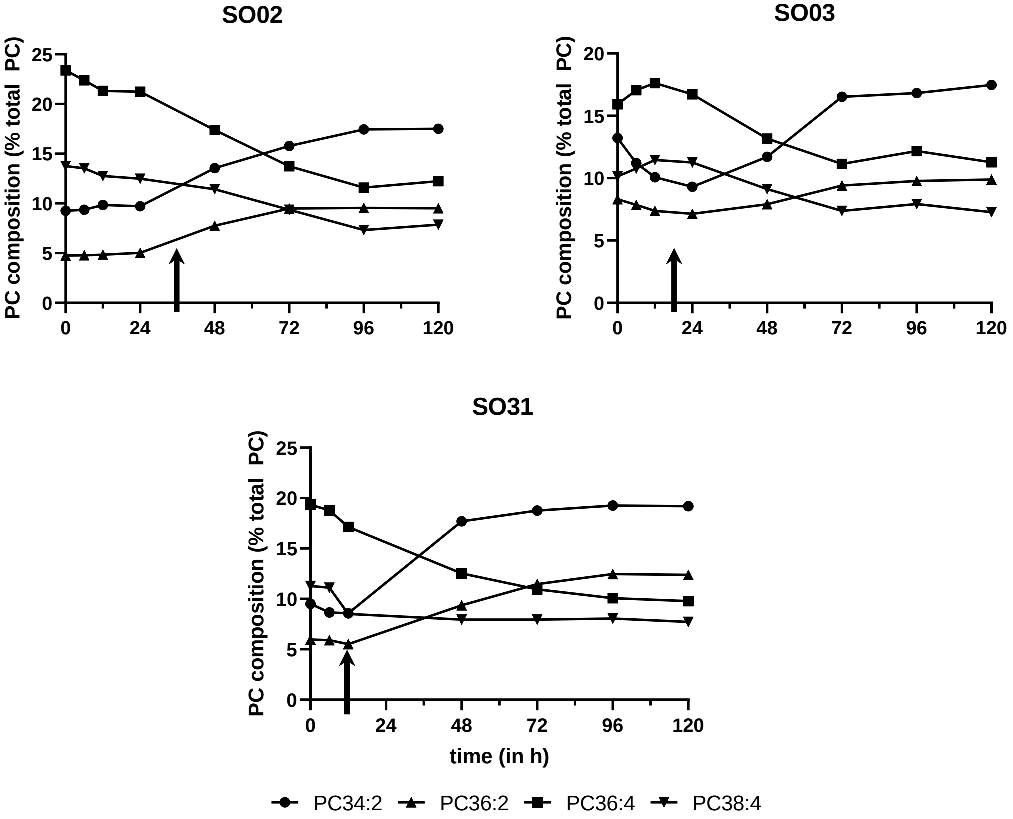

Postnatal adaptations of phosphatidylcholine metabolism in extremely preterm infants: implications for choline and polyunsaturated fatty acid metabolism. Goss K.C.W. “Online Supplementary Material”

Online Supporting Material

Reference

1. Johnson MJ, Pearson F., Emm A., Moyses HE, Leaf AA. Developing a new screening tool for nutritional risk in neonatal intensive care. Acta Paediatr 2015; 104: e90-93.
